# Supplementary material for: Bis(zinc(II)-dipicolylamine)-functionalized sub-2 μm core-shell microspheres for the analysis of N-phosphoproteome
Source: Nat Commun. 2020 Dec 4;11:6226. doi: 10.1038/s41467-020-20026-1 (PMC7718886; doi:10.1038/s41467-020-20026-1)
Supplement: Supplementary file 1 — Supplementary Information [file 41467_2020_20026_MOESM1_ESM.pdf]

Supplementary Information

Bis(zinc(II)-dipicolylamine)-functionalized sub-2 μm core-shell microspheres for analysis of N-phosphoproteome

Yechen Hu [1,2,3#], Bo Jiang [1,#,] *, Yejing Weng[1,2], Zhigang Sui[1], Baofeng Zhao[1], Yuanbo Chen[1,2], Lukuan Liu[1,2], Qiong Wu[1,2], Zhen Liang[1], Lihua Zhang[1,]* and Yukui Zhang[1]

[1] CAS Key Laboratory of Separation Science for Analytical Chemistry, National Chromatographic R & A Center, Dalian Institute of Chemical Physics, Chinese Academy of Sciences, Dalian 116023, China.

[2] University of Chinese Academy of Sciences, Beijing 100049, China.

[3] School of Pharmacy, Nanjing Medical University, Nanjing, 211166, China.

* Corresponding Author: lihuazhang@dicp.ac.cn. Fax: +86-411-84379720

jiangbo@dicp.ac.cn. Fax: +86-411-84379720

# These authors contributed equally to this paper.

Table of Contents

Supplementary Methods

Supplementary Figures

Supplementary Fig.1 (a) 1H NMR and (b) 31P NMR studies of pHis before and after the addition of DpaZn

Supplementary Fig.2 ESI-MS spectra of **1** (a), **2** (b) and **3** (c)

Supplementary Fig.3 Zoom-in XRD spectrum of SiO_2@DpaZn

Supplementary Fig.4 Workflow of the capture and enrichment strategy using the SiO_2@DpaZn

Supplementary Fig.5Workflow of (a) direct analysis, (b) on-tip and (c) in solution enrichment of N-phosphopeptide ($TS_pHYSIMAR$) by SiO_2@DpaZn

Supplementary Fig.6 Recovery of $TS_pHYSIMAR$ by on-tip and in solution enrichment

Supplementary Fig.7 Mannual-check principle of all the PSMs bearing phosphorylation sites

Supplementary Fig.8 Validation of N-pho sites by MS/MS assignments

Supplementary Fig.9 Assessment of the reproducibility of enrichment procedures

Supplementary Fig.10 Sequence motif analysis of identified N-pho sites from (a) HeLa and (b) HEPG2 lysates

Supplementary Fig.11 Biological process analysis of N-phosphoproteins from HeLa lysates

Supplementary Fig.12 Contact angle and zeta potential of SiO_2-NH_2, SiO_2@Dpa, and SiO_2@DpaZn

Supplementary Fig.13 Typical ITC spectral of DpaZn during titration with a pLys peptide-c

Supplementary Fig.14 The overlap of N-pho sites between the three methods

Supplementary Fig.15 Synthesis of Carboxyl Terminal Dpa (**3**)

Supplementary Fig.16 ^{1}H NMR spectrum of **1**

Supplementary Fig.17 ^{1}H NMR spectrum of **2**

Supplementary Fig.18 ^{1}H NMR spectrum of **3**

Supplementary References

Supplementary Methods

Synthesis of N-phosphorylated Peptides

Potassium phosphoramidate (PPA) was synthesized as Wei and Matthews[1]. Standard peptides were dissolved in 10 mM potassium hydroxide aqua solution at final concentration of 2 mg/mL, then, powdered PPA was added to the solution with final concentration of 100 mg/mL. After incubation at room temperature for 12 h, obtained protein or peptides were purified by RP-HPLC and identified by MALDI-TOF MS. According to our previous work[2, 3], PPA can be used to chemically phosphorylate lysine when the peptide sequence does not contain histidine.

Peptide Desalting by Liquid Chromatography

The obtained peptides were desalted using a home-made trap column (4.6 mm i.d. ×1 cm) packed with 5 μm, 100 Å Venusil XBP C18 silica particles (Bonna-Agela Technology, Tianjin, China) with no-acid mobile phases (A: 2% ACN, B: 98% ACN) at flow rate of 1 mL/min on a Shimadzu DGU-20A5 liquid chromatography with a diode array detector (Shimadzu DGU-20A5, Kyoto, Japan). The collected peptides were lyophilized dry at room temperature and stored at -80°C for further use.

Peptide Desalting by High pH Reversed Phase Tips

Home-made high pH reversed phase microcolumns (HpH-RP Tips) were prepared by packing 1 mg 5 μm 100 Å Durashell C18 silica particles (Bonna-Agela Technology, Tianjin, China) into a 20 μL micropipet tip through centrifugation at 5000 rpm. Then, microcolumns were equilibrated with 10 μL phase A (10 mM ammonium acetate in 2% ACN, pH 10). After sampling, columns were washed with 10 μL phase A. Finally, samples were eluted with 10 μL phase B (10 mM ammonium acetate in 80% ACN, pH 10). The eluates were deposited onto a MALDI-MS plate directly or kept in -80°C for further use.

Stability of N-phosphopeptide under Votex and Ultrasonic Treatment

300 μg of standard peptide 1297 (TSHYSIMAR) was dissolved in 50 mM phosphate-buffered saline (PBS), and divided into 3 equal parts. Each part reacted with different dimethyl labeling reagents: 4% formaldehyde-H_2 with 0.6 M sodium cyanoborohydride, 4% formaldehyde-D_2 with 0.6 M sodium cyanoborohydride and 4% formaldehyde-^{13}C-D_2 with 0.6 M sodium cyanoborodeuteride, to form $(CH_3)_2$-TSHYSIMAR (pep-L), $(CHD_2)_2$-TSHYSIMAR (pep-M), and $(^{13}CD_3)_2$-TSHYSIMAR (pep-H), respectively. After labeling at room temperature for 1 h, the reaction was terminated with 0.1% $NH_3 \cdot H_2O$, and desalted by liquid chromatography. The lyophilized peptides were incubated with PPA to generate

N-phosphopeptides: $(CH_3)_2$-TSH$_p$YSIMAR (NPpep-L), $(CHD_2)_2$-TSH$_p$YSIMAR (NPpep-M), and $(^{13}CD_3)_2$-TSH$_p$YSIMAR (NPpep-H), respectively.

The 3 N-phosphopeptides were desalted through HpH-RP Tips, and applied to three operations respectively: ① 100 μg NPpep-L were kept with no operation, ②100 μg NPpep-M were treated with votex; ③ 100 μg NPpep-H were treated with ultrasonic. After incubated at room temperature for 0 min, 2 min, 5 min, 10 min, 20 min, 0.5 μL of each peptide was mixed and measured by MALDI-TOF MS. Three sets of parallel experiments were performed simultaneously.

Free Solution Enrichment of Phosphopeptides by SiO_2@DpaZn

Peptides were dissolved in 200 μL 80% ACN (20 mM HEPES, pH 7.7). After incubation with 1 mg SiO_2@DpaZn at room temperature for 30 min, total volume of 400 μL 50% ACN, 400 μL 0.001% $NH_3\cdot H_2O$, 400 μL 0.005% $NH_3\cdot H_2O$ comprised washing steps, respectively. The total washing time is 30 min. Finally, phosphopeptides were eluted by incubating with 100 μL 0.1% $NH_3\cdot H_2O$ for 15 min and analyzed by MALDI-TOF MS.

Comparison of Fast On-tip Enrichment and In-solution Enrichment of N-phosphopeptides

30 μg of standard peptide (TSHYSIMAR) was dissolved in 50 mM phosphate-buffered saline (PBS), and divided into 3 equal parts. Each part reacted with different dimethyl labeling reagents: 4% formaldehyde-H_2 with 0.6 M sodium cyanoborohydride, 4% formaldehyde-D_2 with 0.6 M sodium cyanoborohydride and 4% formaldehyde-^{13}C-D_2 with 0.6 M sodium cyanoborodeuteride, to form $(CH_3)_2$-TSHYSIMAR (pep-L), $(CHD_2)_2$-TSHYSIMAR (pep-M), and $(^{13}CD_3)_2$-TSHYSIMAR (pep-H), respectively. After labeling at room temperature for 1 h, the reaction was terminated with 0.1% $NH_3\cdot H_2O$, and desalted by liquid chromatography. The lyophilized peptides were incubated with PPA to generate N-phosphopeptides: $(CH_3)_2$-TSH$_p$YSIMAR (NPpep-L), $(CHD_2)_2$-TSH$_p$YSIMAR (NPpep-M), and $(^{13}CD_3)_2$-TSH$_p$YSIMAR (NPpep-H), respectively.

The 3 N-phosphopeptides were desalted through HpH-RP Tips, and applied to three parallel operations respectively: ① 5 μg NPpep-L were kept in -80°C as a control, ② 2.5 μg NPpep-M were quick enriched by SiO_2@DpaZn tip and ③ 2.5 μg NPpep-H were enriched by SiO_2@DpaZn in free solution with regular operation.

Elutes from the 3 operations were merged and analyzed by LC-MS. The recovery (R) of NPpep was described by the area of extract ion chromatography (XIC) as follow:

R (NPpep-X) = [Area(NPpep-X)$_{after}$ / Area (NPpep-X)$_{before}$] * [Area (NPpep-L)$_{before}$ / Area (NPpep-L)$_{after}$], where Area (NPpep-X)$_{before}$ and Area (NPpep-X)$_{after}$ were the XIC area of NPpep-X before and after enrichment.

High pH-RP Fractionation

Shimadzu DGU-20A5 liquid chromatography carrying a home-packed high-pH C18 column 5 μm 100 Å Durashell C18 silica particles (2.1 mm i.d. × 150 mm) was used for phosphopeptide separation. The mobile phases were prepared and adjusted to pH 10 with 28% $NH_3 \cdot H_2O$ as follows. Buffer A: 2% (v/v) ACN with 10 mM ammonium acetate, buffer B: 80% (v/v) ACN with 10 mM ammonium acetate. With a flow rate of 0.3 mL/min, 80 min-separation gradient (70 min from 0 to 45% B, 5 min from 45 to 100% B, and maintain at 100% B for 5min) was applied. Peptides were collected from every-minute elution time window respectively from 10 to 69 min (Window1: 10min, Window2: 11min, … , Window60: 69min), and 6 equal interval windows were merged into total 10 fractions (e.g. Fraction1: 10 min + 20 min + 30 min + 40 min + 50 min + 60 min). Every fraction was lyophilized and resolved in water for MS analysis. For mammal lysate, 20 fractions were collected for MS analysis.

PSM Manual Selected Roles

For all the PSMs: 1) Controlling the FDR at PSMs level to be <1%; 2) Setting the cut-off value of ion score to be 20; For all spectra of a peptide, 3) the phosphorylation site was determined if their scores were the same and point to the same residues; 4) if their scores were different, the site with the highest score was the phosphorylation site; 5) otherwise, the exact phosphorylation sites were indeterminate.

Comparison of SiO_2@DpaZn, SiO_2@Dpa and SiO_2@NH_2

30 μg of standard peptide 1297 (TSHYSIMAR) was dissolved in 50 mM phosphate-buffered saline (PBS), and divided into 3 equal parts. Each part reacted with different dimethyl labeling reagents: 4% formaldehyde-H_2 with 0.6 M sodium cyanoborohydride, 4% formaldehyde-D_2 with 0.6 M sodium cyanoborohydride and 4% formaldehyde-^{13}C-D_2 with 0.6 M sodium cyanoborodeuteride, to form $(CH_3)_2$-TSHYSIMAR (pep-L), $(CHD_2)_2$-TSHYSIMAR (pep-M), and $(^{13}CD_3)_2$-TSHYSIMAR (pep-H), respectively. After labeling at room temperature for 1 h, the reaction was terminated with 0.1% $NH_3 \cdot H_2O$, and desalted by liquid chromatography. The lyophilized peptides were incubated with PPA to generate N-phosphopeptides: $(CH_3)_2$-TSH$_p$YSIMAR (NPpep-L), $(CHD_2)_2$-TSH$_p$YSIMAR (NPpep-M), and $(^{13}CD_3)_2$-TSH$_p$YSIMAR (NPpep-H), respectively.

The 3 N-phosphopeptides were desalted through HpH-RP Tips, and applied to three parallel operations respectively: ① 10 μg NPpep-L were mixed with 10 μg interference peptide (TGIFKSAR) and 200 μg SiO_2@DpaZn in 55% ACN; ②10 μg NPpep-M were mixed with 10 μg interference peptide (TGIFKSAR) and 200 μg SiO_2@Dpa in 55% ACN; ③ 10 μg NPpep-H were mixed with 10 μg interference peptide (TGIFKSAR) and 200 μg SiO_2@NH_2 in 55% ACN. After incubated at room temperature for 30 min, 80% ACN and 20 mM HEPES were performed to wash the microspheres. Finally, the peptides were eluted in 1% $NH_3 \cdot H_2O$ and mixed for ESI-MS/MS analysis.

Step Elution of Phosphopeptides

1 mg SiO_2@DpaZn in 80% ACN (20 mM HEPES, pH 7.7) was packed into a 20 μL micropipet tip by centrifugation at 5000 rpm for 10 min. Dissolved in 80% ACN (20 mM HEPES, pH 7.7), 2.5 μg standard N-phosphopeptide (TSpHYSIMAR) and 25 μg β-casein digests were load onto the tip by centrifugation at 5000 rpm for 3 min. 50 μL 50% ACN (20 mM HEPES, pH 7.7), 15 μL 0.005% $NH_3 \cdot H_2O$, 15 μL 0.01% $NH_3 \cdot H_2O$, and 15 μL 0.1% $NH_3 \cdot H_2O$ were set as washing-elution steps, respectively. Elutes from every step were analyzed by MALDI-TOF MS.

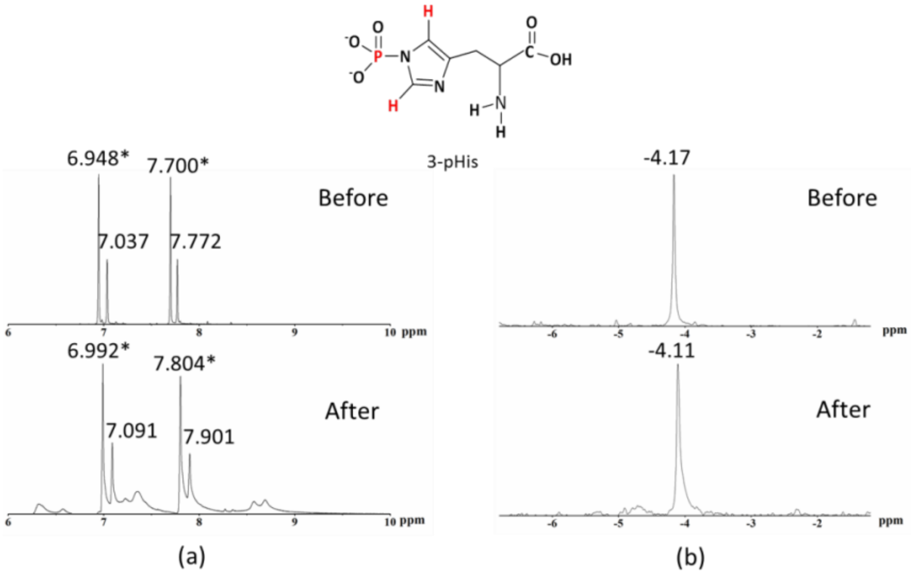

Supplementary Fig. 1 (a) 1H NMR and (b) 31P NMR studies of pHis before and after the addition of DpaZn. Asterisks indicate the dephosphorylated counterpart of pHis.

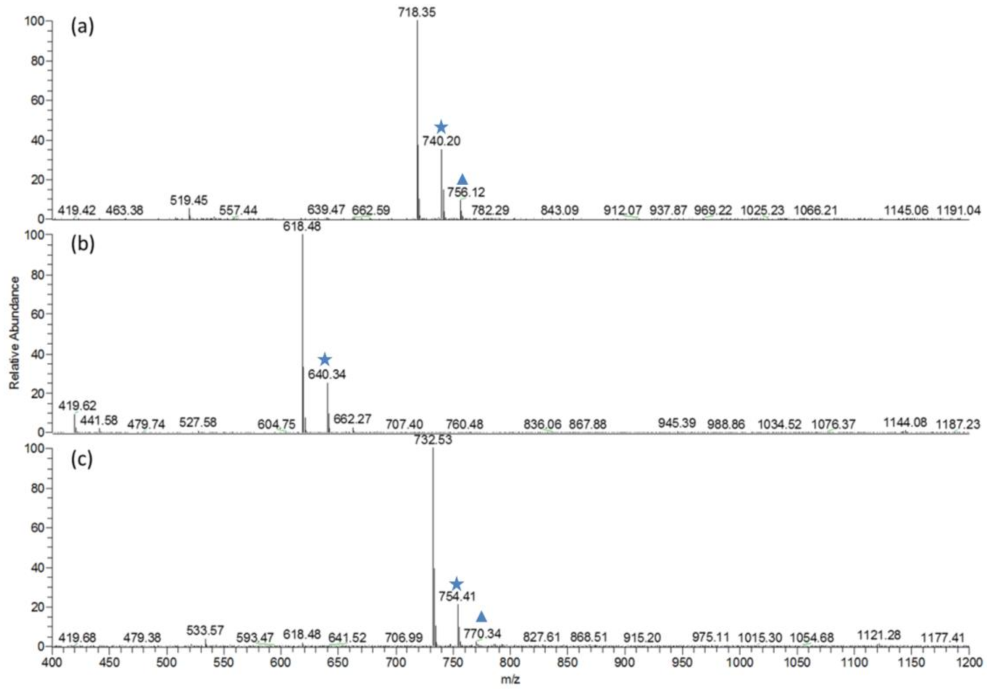

Supplementary Fig. 2 ESI-MS spectra of **1** (a), **2** (b) and **3** (c). Stars and triangles represent $[M+Na]^+$ and $[M+K]^+$, respectively.

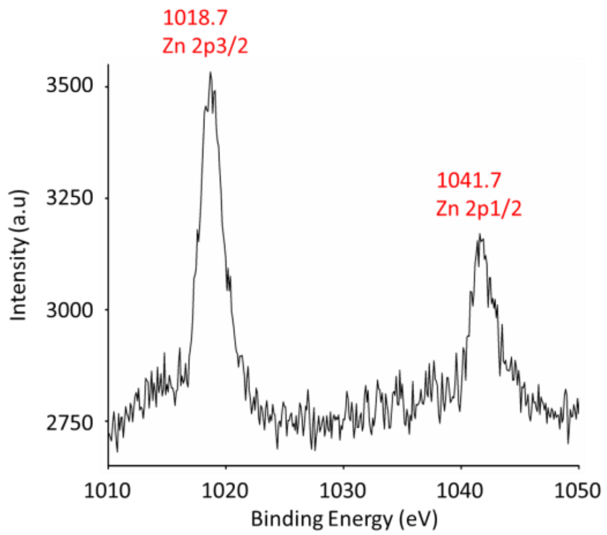

Supplementary Fig. 3 Zoom-in XRD spectrum of SiO_2@DpaZn. The two peaks of Zn(II) were labelled in red.

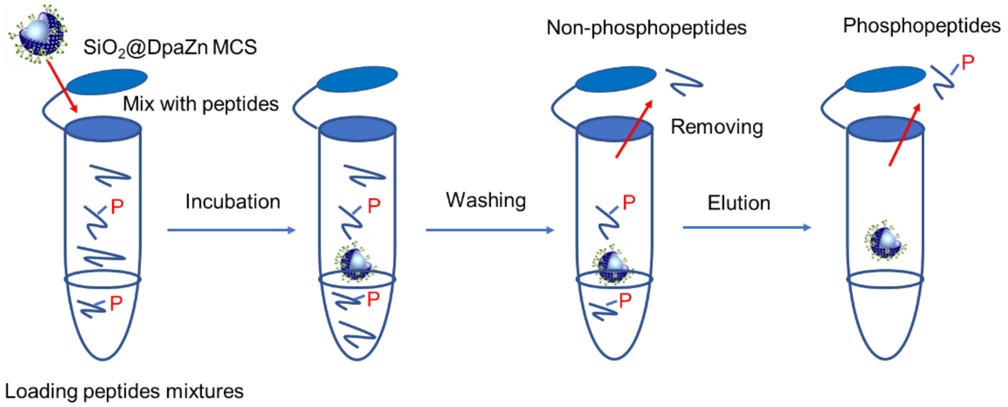

Supplementary Fig. 4 Workflow of the capture and enrichment strategy using the SiO_2@DpaZn.

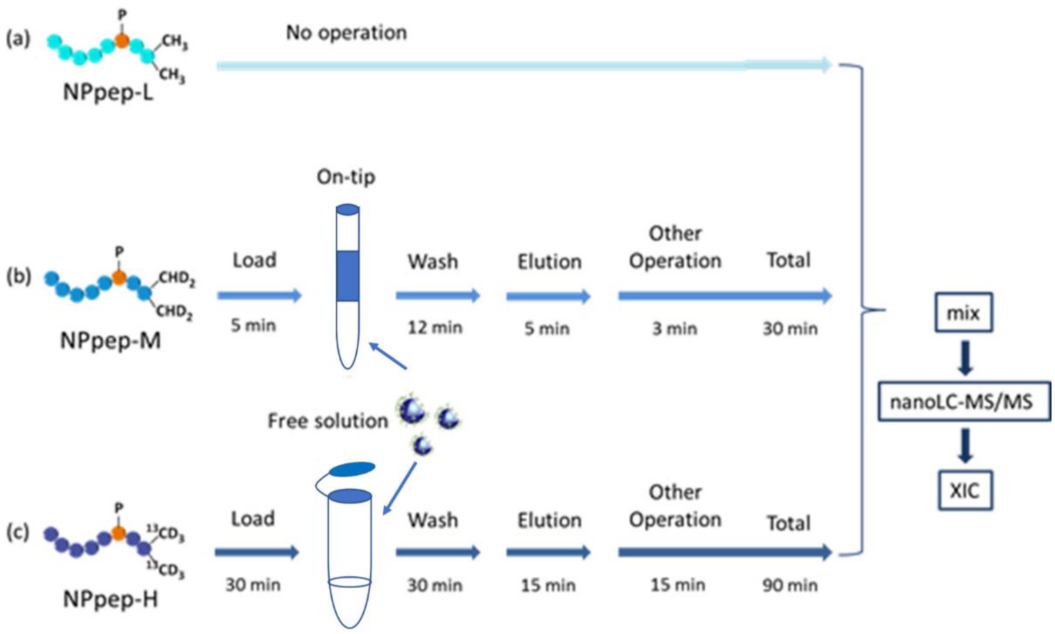

Supplementary Fig. 5 Workflow of (a) Direct analysis, (b) on-tip and (c) in solution enrichment of N-phosphopeptide ($TS_pHYSIMAR$) by SiO_2@DpaZn.

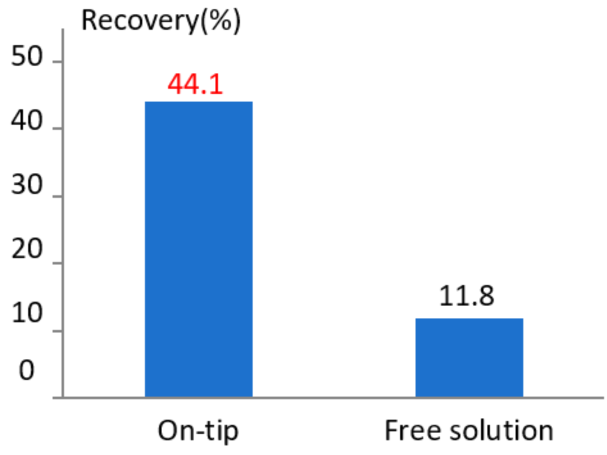

Supplementary Fig. 6 Recovery of $TS_pHYSIMAR$ by on-tip and in solution enrichment.

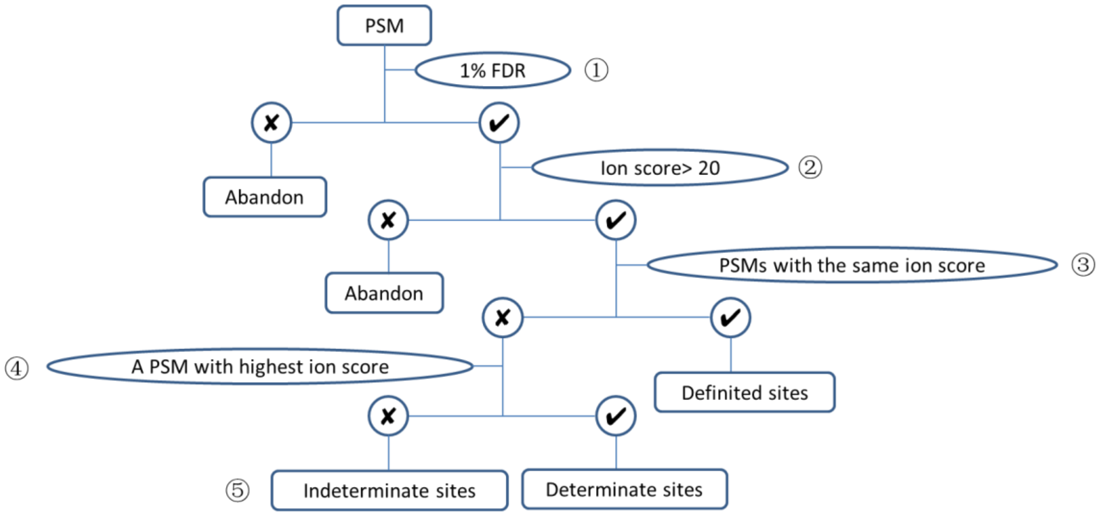

Supplementary Fig. 7 Mannual-check principle of all the PSMs bearing phosphorylation sites. For all the PSMs: 1) Controlling the FDR at PSMs level to be <1%; 2) Setting the cut-off value of ion score to be 20; For all spectra of a peptide, 3) the phosphorylation site was determined if their scores were the same and point to the same residues; 4) if their scores were different, the site with the highest score was the phosphorylation site; 5) otherwise, the exact phosphorylation sites were indeterminate.

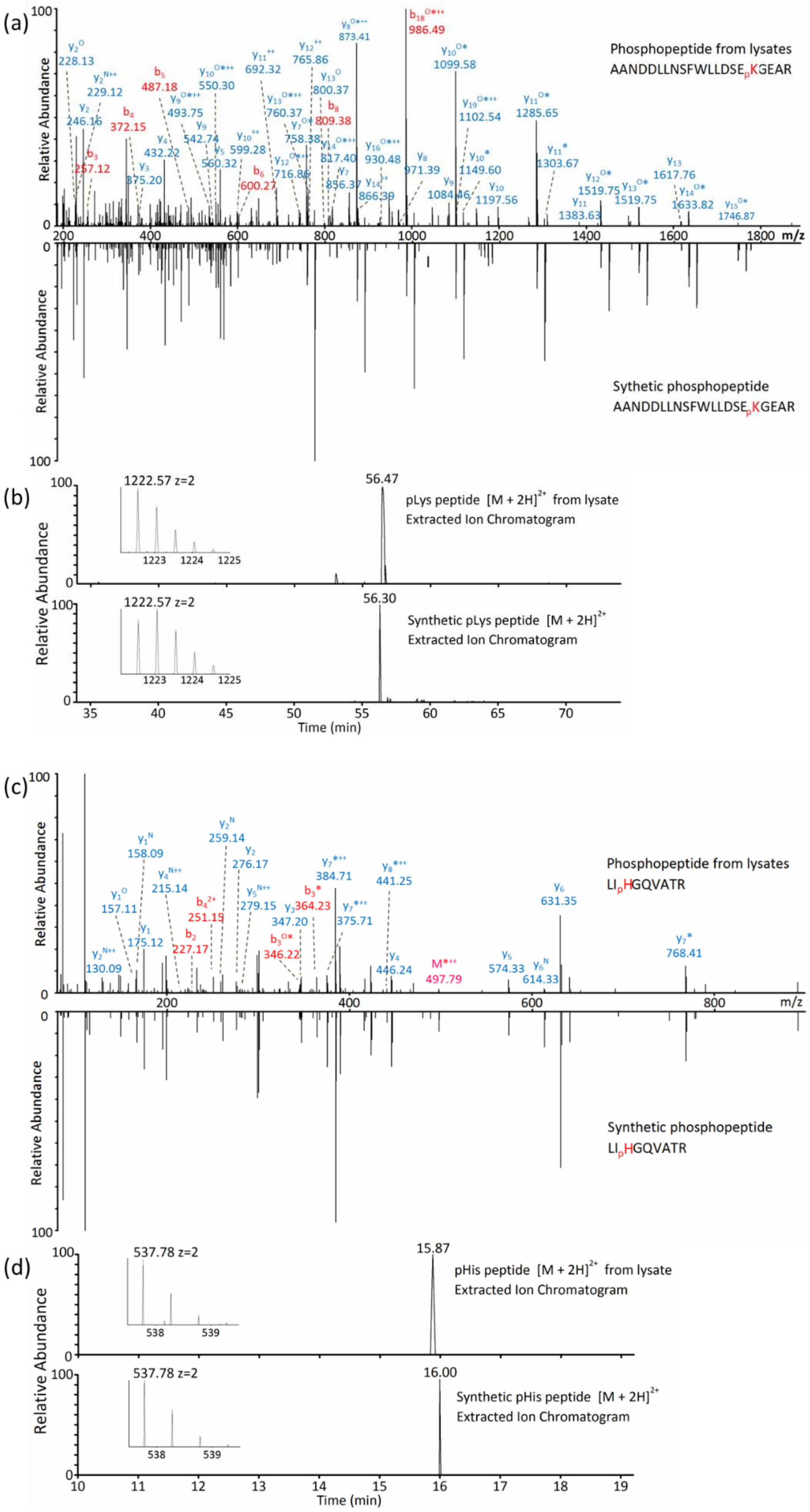
(a)
Phosphopeptide from lysates
AANDDLLNSFWLLDSEpKGEAR
Sythetic phosphopeptide
AANDDLLNSFWLLDSEpKGEAR
Relative Abundance
m/z
(b)
1222.57 z=2
56.47
pLys peptide [M + 2H]2+ from lysate
Extracted Ion Chromatogram
1222.57 z=2
56.30
Synthetic pLys peptide [M + 2H]2+
Extracted Ion Chromatogram
Relative Abundance
Time (min)
(c)
Phosphopeptide from lysates
LIpHGQVATR
Synthetic phosphopeptide
LIpHGQVATR
Relative Abundance
m/z
(d)
537.78 z=2
15.87
pHis peptide [M + 2H]2+ from lysate
Extracted Ion Chromatogram
537.78 z=2
16.00
Synthetic pHis peptide [M + 2H]2+
Extracted Ion Chromatogram
Relative Abundance
Time (min)

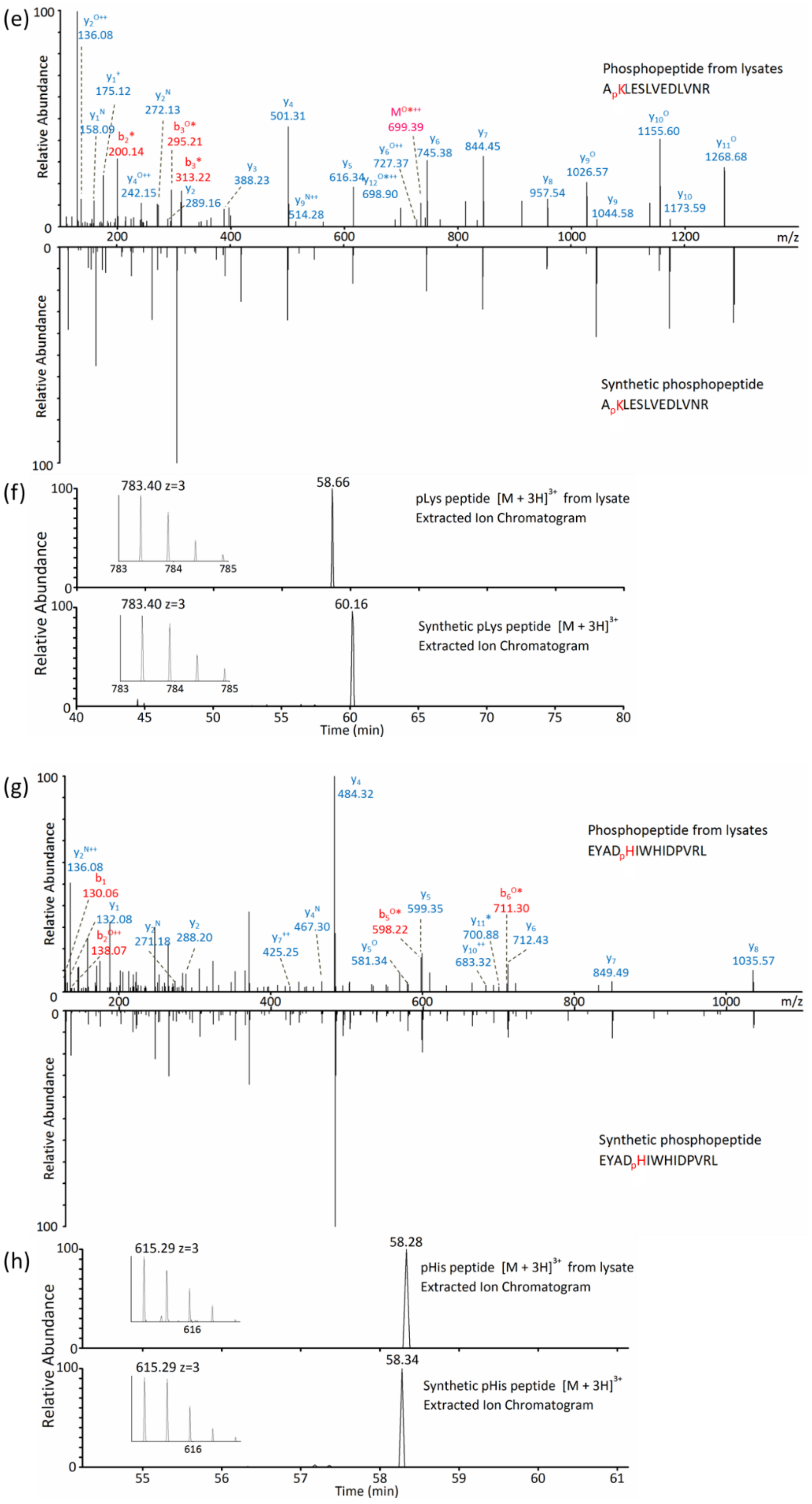
(e)
Relative Abundance
Phosphopeptide from lysates
A pK LESLVEDLVNR
Synthetic phosphopeptide
A pK LESLVEDLVNR
136.08
175.12
272.13
158.09
200.14
295.21
313.22
242.15
289.16
388.23
501.31
514.28
616.34
699.39
727.37
698.90
745.38
844.45
957.54
1026.57
1044.58
1155.60
1173.59
1268.68
m/z
(f)
783.40 z=3
58.66
pLys peptide [M + 3H]3+ from lysate
Extracted Ion Chromatogram
60.16
Synthetic pLys peptide [M + 3H]3+
Extracted Ion Chromatogram
Time (min)
(g)
Phosphopeptide from lysates
EYAD pH IWHIDPVRL
Synthetic phosphopeptide
EYAD pH IWHIDPVRL
484.32
136.08
130.06
132.08
138.07
271.18
288.20
425.25
467.30
581.34
598.22
599.35
683.32
700.88
711.30
712.43
849.49
1035.57
m/z
(h)
615.29 z=3
58.28
pHis peptide [M + 3H]3+ from lysate
Extracted Ion Chromatogram
58.34
Synthetic pHis peptide [M + 3H]3+
Extracted Ion Chromatogram
Time (min)

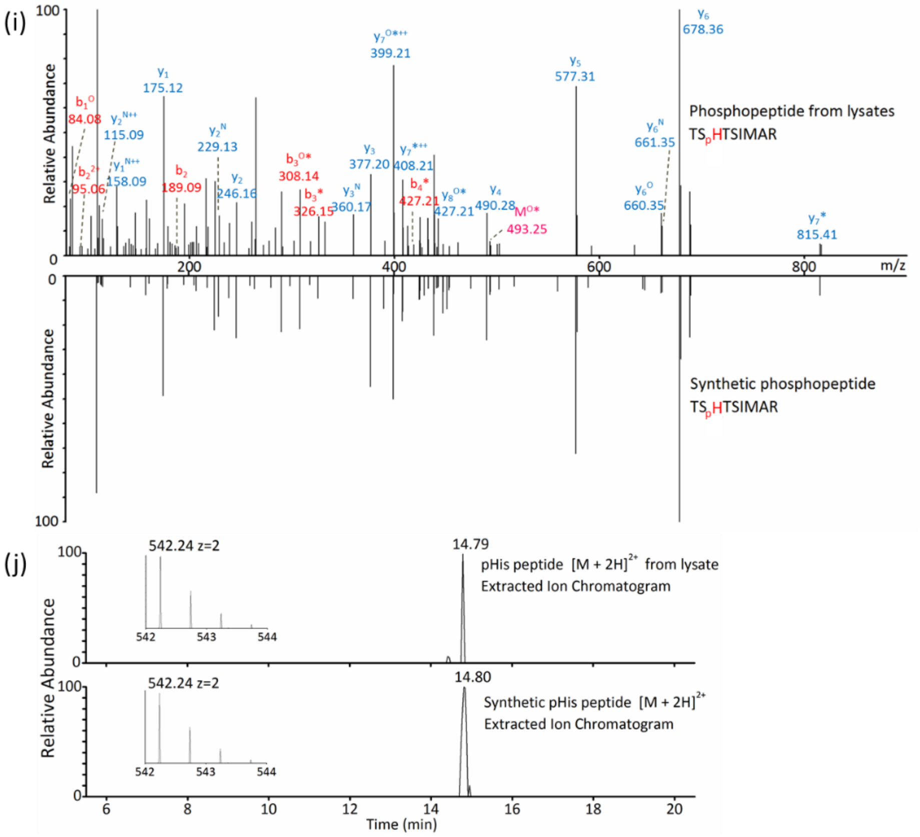

Supplementary Fig. 8 Validation of N-pho sites by MS/MS assignments and HPLC elution experiments. MS/MS spectra and extracted ion chromatograms of the endogenous *E. coli* pLys peptide (AANDDLLNSFWLLDSE$_p$KGEAR) (a) and (b), pHis peptide (LI$_p$HGQVATR) (c) and (d), pLys peptide (A$_p$KLESLVEDLVNR) (e) and (f), pHis peptide (EYAD$_p$HIWHIDPVRL) (g) and (h), and pHis peptide (TS$_p$HTSIMAR) (i) and (j) annotated with the major matched b- and y-type ions indicated. The synthetic peptides are shown in the mirror image. The superscript asterisks, N and O indicate the dephosphorylated (-79.97 Da), deamination (-17.03Da) and dehydrated (-18.01 Da) ions, respectively.

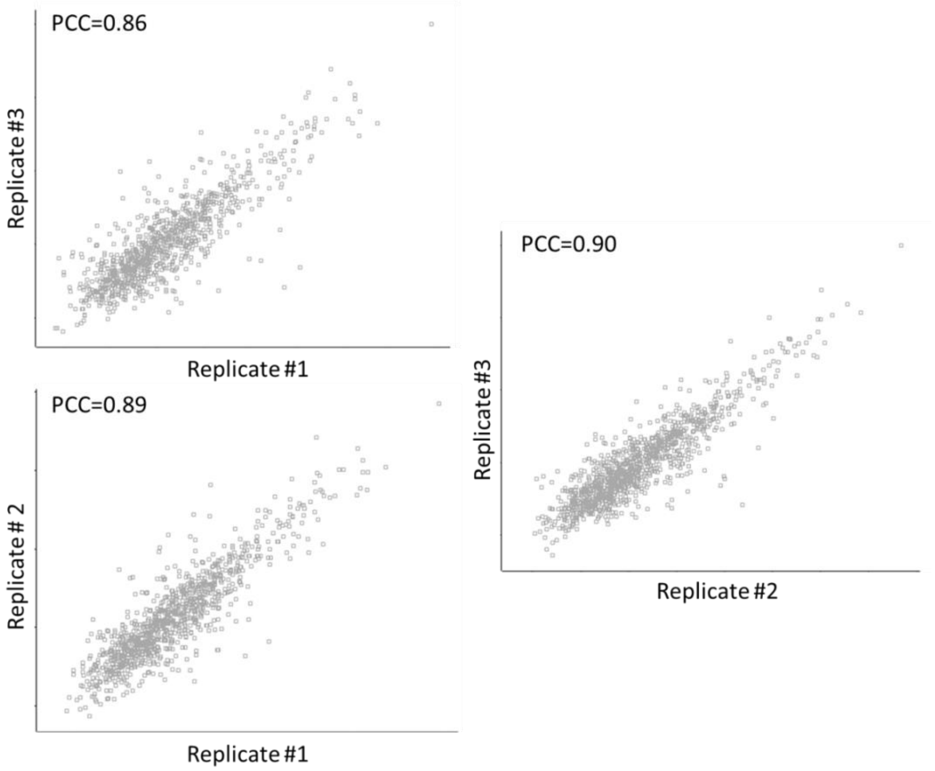

Supplementary Fig. 9 Assessment of the reproducibility of enrichment procedures.

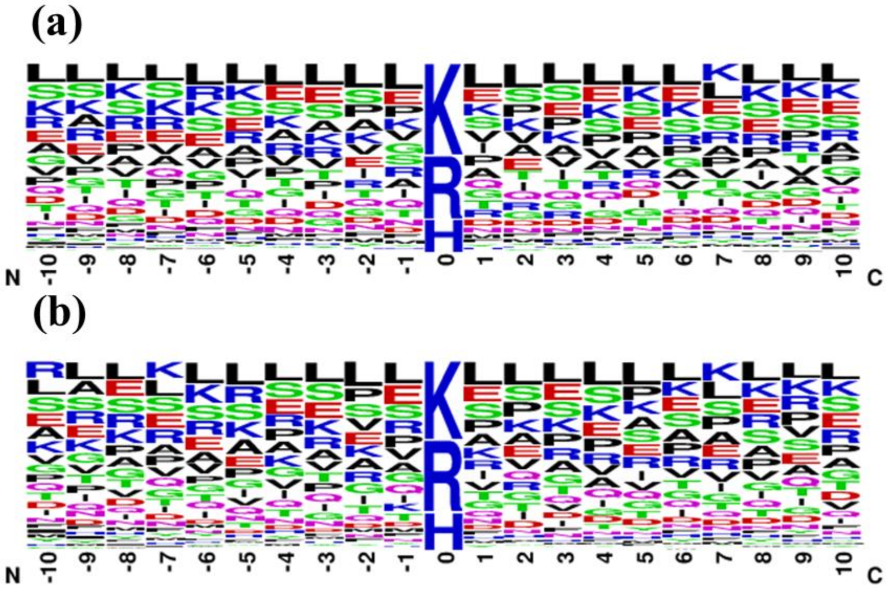

Supplementary Fig. 10 Sequence motif analysis of identified N-pho sites from (a) HeLa and (b) HEPG2 lysates using WebLogo.

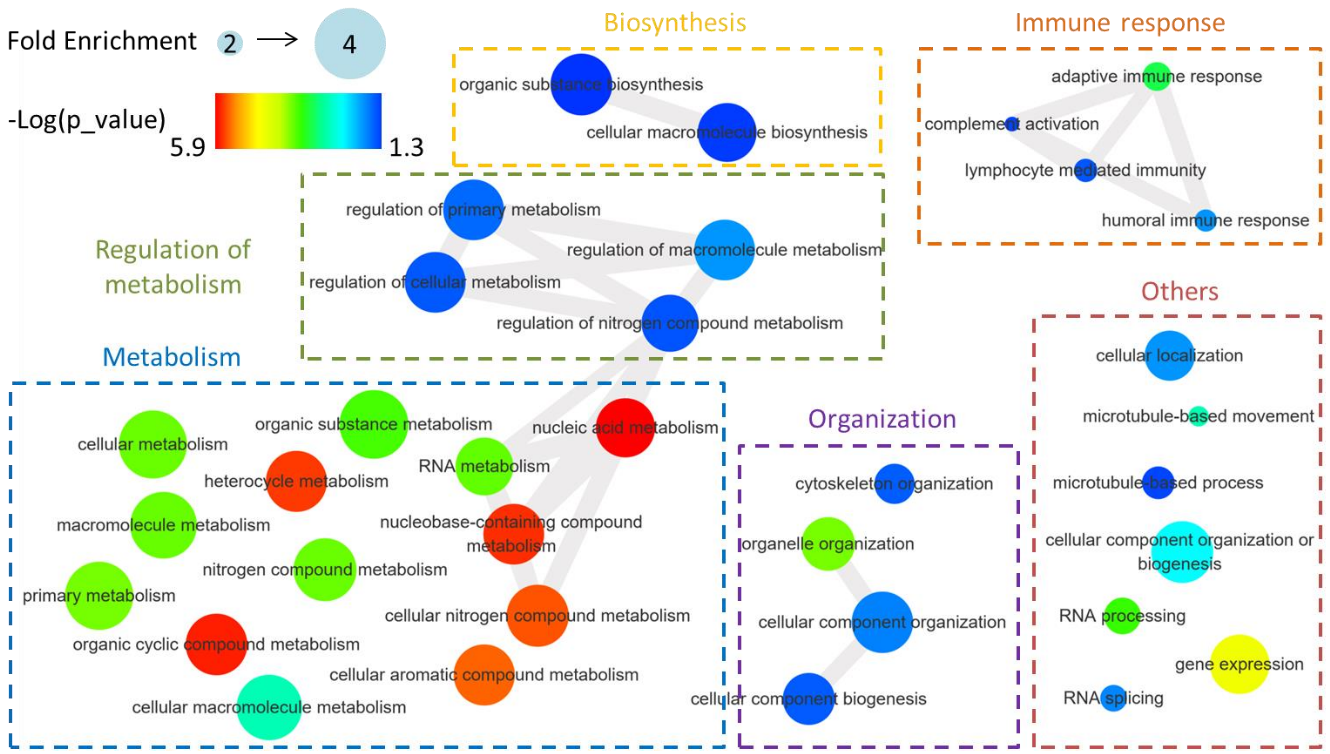

Supplementary Fig. 11 Biological process analysis of N-phosphoproteins from HeLa lysates. The color bar depicts the Log (Fisher's exact test, with the Benjamini–Hochberg false discovery rate (FDR) <1% for multiple testing) and the size stands for the fold of enrichment. We used PANTHER 15.0 to perform Gene Ontology enrichment analysis and the biological processes were summarized in REViGO and visualised using Cytoscape 3.7.2.

Microsphere		Contact angle（°）	Zeta potential（mV）
$SiO_2@NH_2$	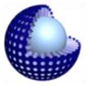	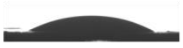 29.0	42.7
$SiO_2@Dpa$	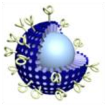	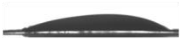 23.3	-44.9
$SiO_2@DpaZn$	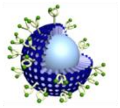	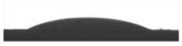 21.8	56.5

Supplementary Fig. 12 Contact angle and Zeta potential of SiO_2-NH_2, SiO_2@Dpa, and SiO_2@DpaZn.

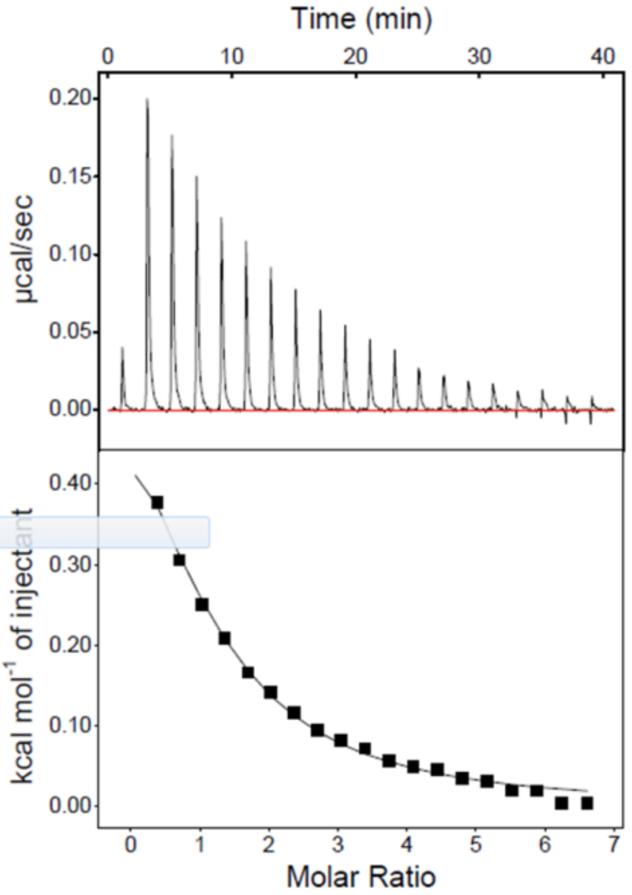

Supplementary Fig. 13 Typical ITC spectral of DpaZn during titration with a pLys peptide.

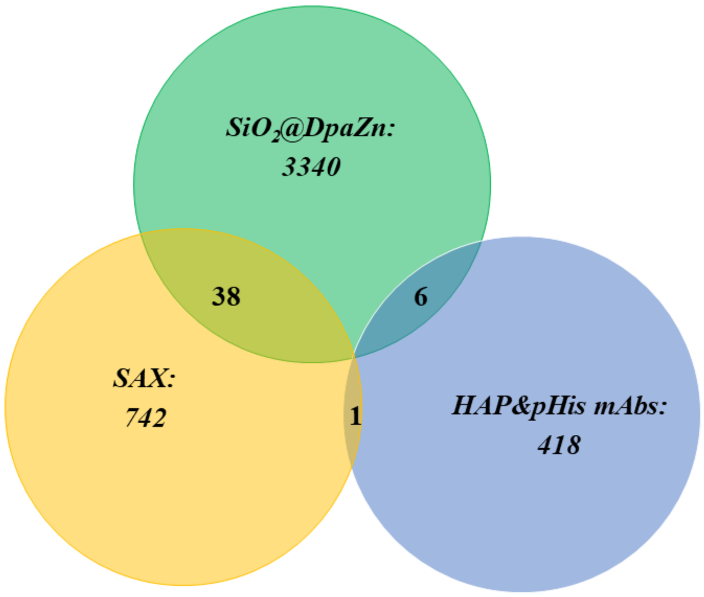

Supplementary Fig. 14 The overlap of N-pho sites between the three methods.

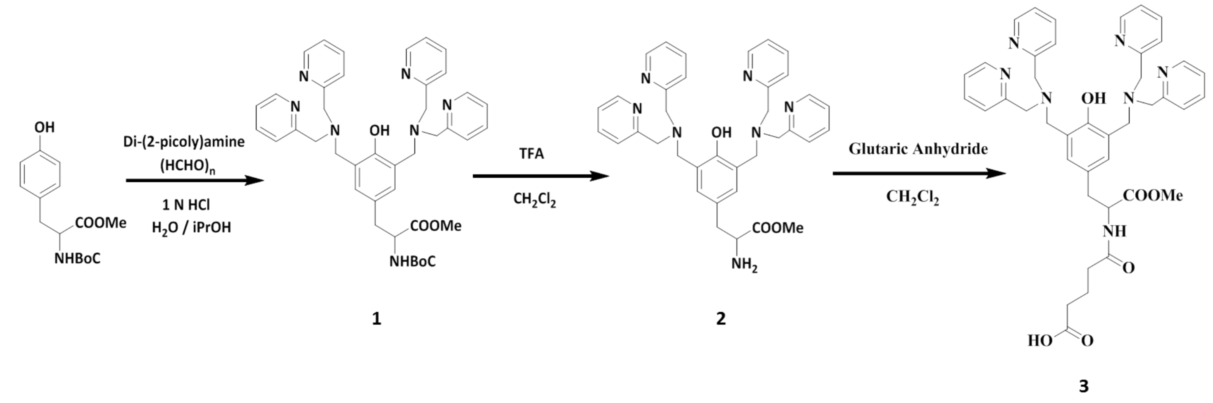

Supplementary Fig. 15 Synthesis of Carboxyl Terminal Dpa (**3**).

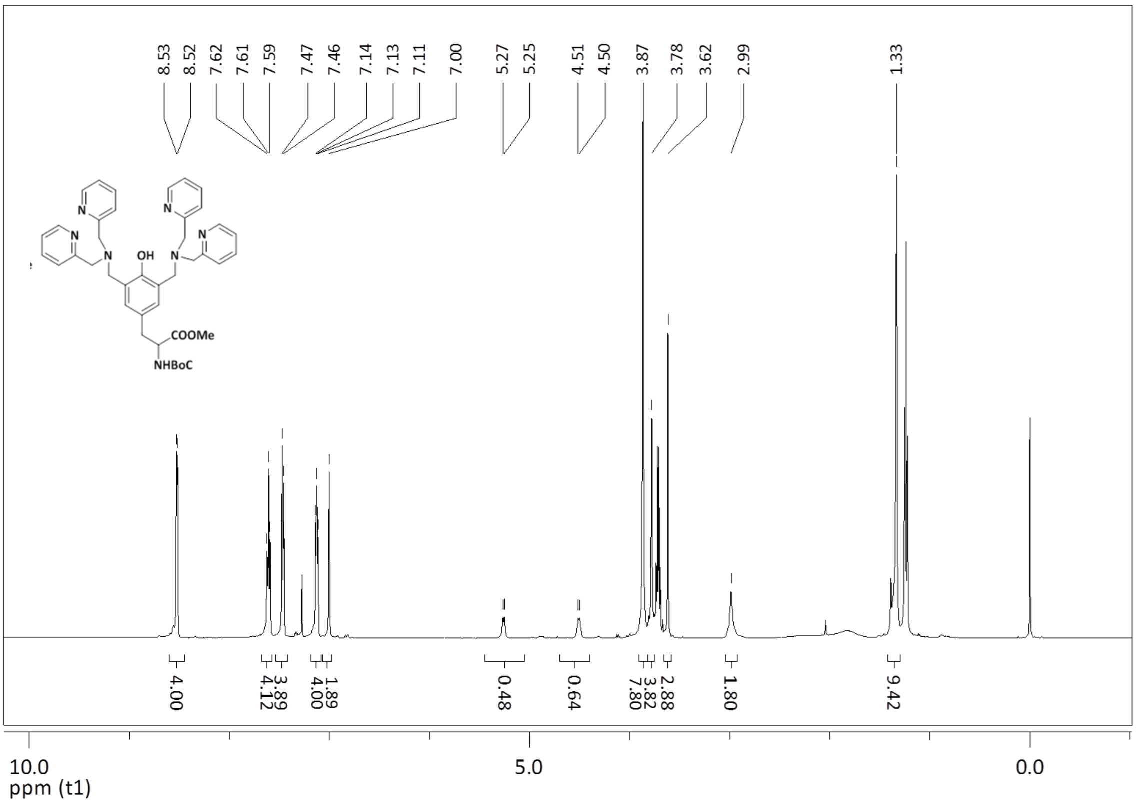

Supplementary Fig. 16 ^{1}H NMR spectrum of **1**.

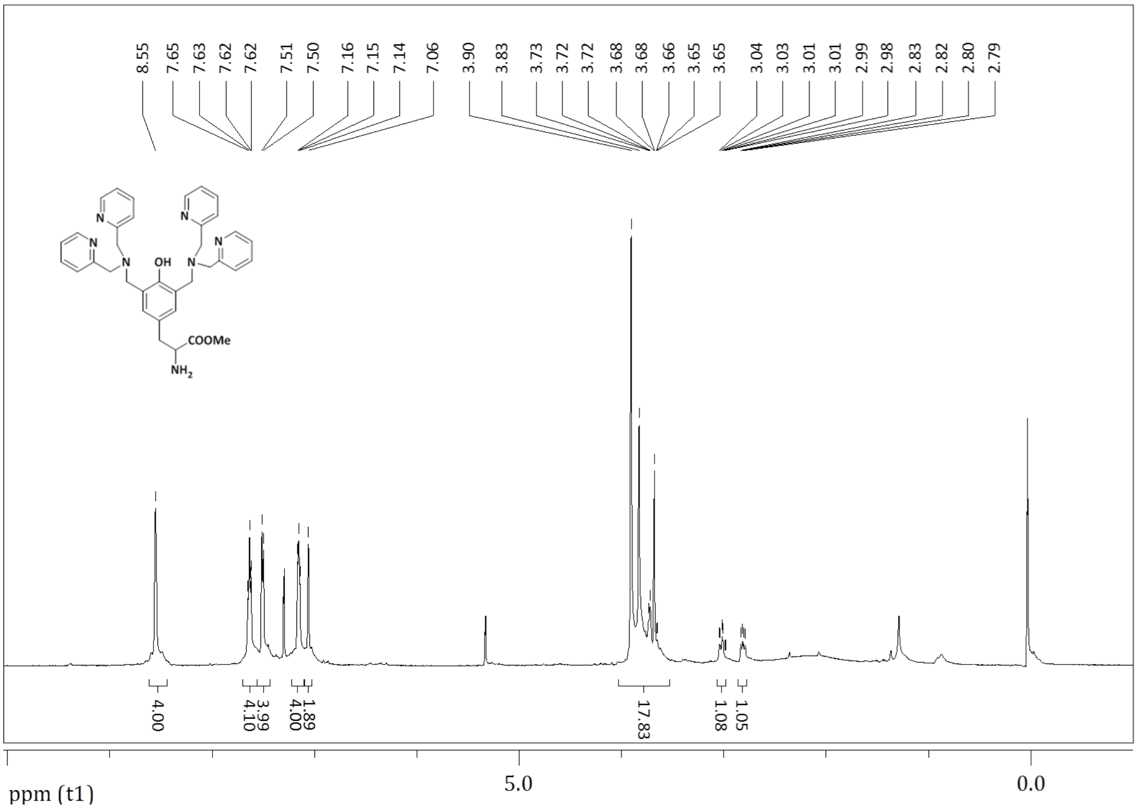

Supplementary Fig. 17 ^{1}H NMR spectrum of **2**.

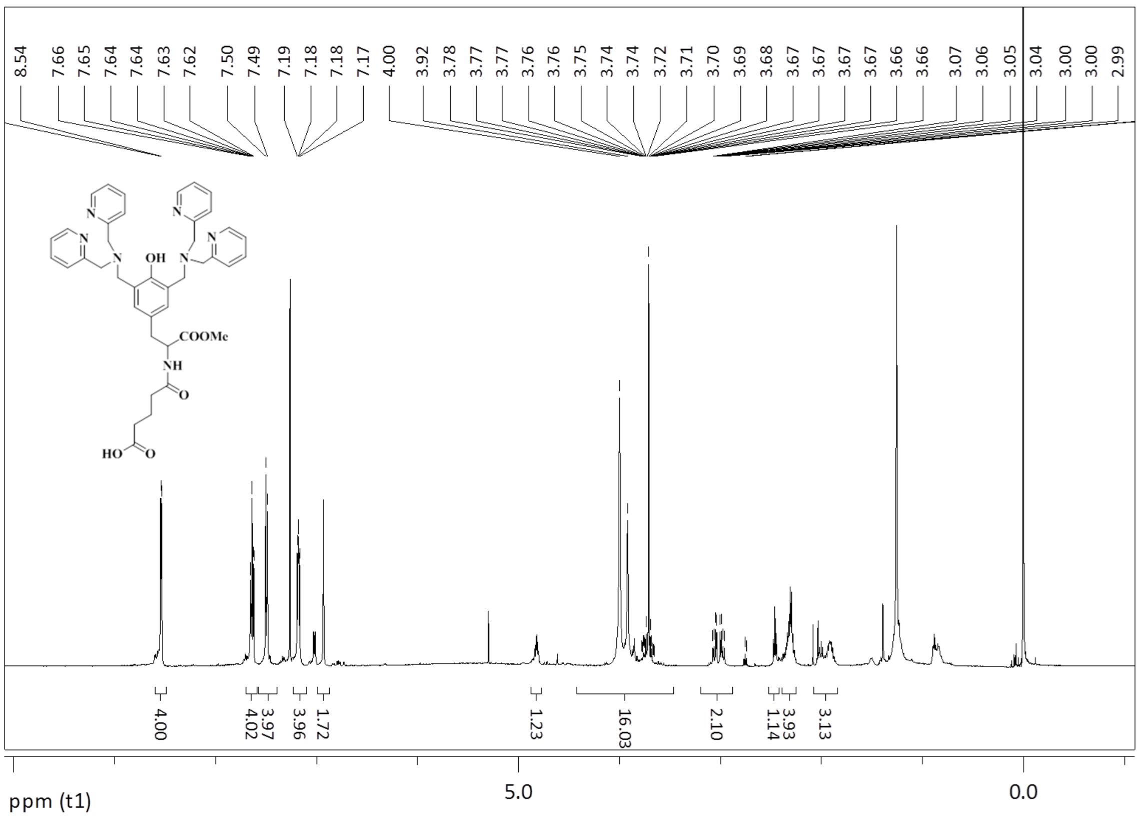

Supplementary Fig. 18 ^{1}H NMR spectrum of **3**.

Supplementary References

1 Wei, Y. F. & Matthews, H. R. A filter-based protein kinase assay selective for alkali-stable protein phosphorylation and suitable for acid-labile protein phosphorylation. *Anal. Chem.* **190**, 188-192 (1990).

2 Hu, Y. C. *et al.* Isolation and identification of phosphorylated lysine peptides by retention time difference combining dimethyl labeling strategy. *Sci China Chem* **62**, 708-712 (2019).

3 Hu, Y. C. *et al.* Cleavable hydrophobic derivatization strategy for enrichment and identification of phosphorylated lysine peptides. *Anal Bioanal Chem* **411**, 4159-4166 (2019).
