# Supplementary material for: Bis(zinc(II)-dipicolylamine)-functionalized sub-2 μm core-shell microspheres for the analysis of N-phosphoproteome
Source: Nat Commun. 2020 Dec 4;11:6226. doi: 10.1038/s41467-020-20026-1 (PMC7718886; doi:10.1038/s41467-020-20026-1)
Supplement: Supplementary file 3 — Descriptions of Additional Supplementary Files [file 41467_2020_20026_MOESM3_ESM.pdf]

Descriptions of Additional Supplementary Files

Supplementary dataset 1

Description: Phosphorylation results of LB cultured E.coli. 27 N-pho sites, containing 15 pHis, 8 pLys and 4 pArg, were identified.

Supplementary dataset 2

Description: Phosphorylation results of different carbon source cultured E.coli. 99 N-pho sites, containing 19 pHis, 38 pLys and 42 pArg, were identified.

Supplementary dataset 3

Description: Phosphorylation results of HeLa. 3384 N-pho sites, containing 611 pHis, 1618 pLys and 1155 pArg, were identified]. T
