# Supplementary material for: Bis(zinc(II)-dipicolylamine)-functionalized sub-2 μm core-shell microspheres for the analysis of N-phosphoproteome
Source: Nat Commun. 2020 Dec 4;11:6226. doi: 10.1038/s41467-020-20026-1 (PMC7718886; doi:10.1038/s41467-020-20026-1)
Supplement: Supplementary file 7 — Reporting Summary [file 41467_2020_20026_MOESM7_ESM.pdf]

nature research

Corresponding author(s): Lihua Zhang

Last updated by author(s): Oct 26, 2020

Reporting Summary

Nature Research wishes to improve the reproducibility of the work that we publish. This form provides structure for consistency and transparency in reporting. For further information on Nature Research policies, see our Editorial Policies and the Editorial Policy Checklist.

Statistics

For all statistical analyses, confirm that the following items are present in the figure legend, table legend, main text, or Methods section.

n/a	Confirmed	
[x]	[ ]	The exact sample size (*n*) for each experimental group/condition, given as a discrete number and unit of measurement
[ ]	[x]	A statement on whether measurements were taken from distinct samples or whether the same sample was measured repeatedly
[ ]	[x]	The statistical test(s) used AND whether they are one- or two-sided<br>*Only common tests should be described solely by name; describe more complex techniques in the Methods section.*
[x]	[ ]	A description of all covariates tested
[x]	[ ]	A description of any assumptions or corrections, such as tests of normality and adjustment for multiple comparisons
[ ]	[x]	A full description of the statistical parameters including central tendency (e.g. means) or other basic estimates (e.g. regression coefficient) AND variation (e.g. standard deviation) or associated estimates of uncertainty (e.g. confidence intervals)
[ ]	[x]	For null hypothesis testing, the test statistic (e.g. *F*, *t*, *r*) with confidence intervals, effect sizes, degrees of freedom and *P* value noted<br>*Give P values as exact values whenever suitable.*
[x]	[ ]	For Bayesian analysis, information on the choice of priors and Markov chain Monte Carlo settings
[x]	[ ]	For hierarchical and complex designs, identification of the appropriate level for tests and full reporting of outcomes
[ ]	[x]	Estimates of effect sizes (e.g. Cohen's *d*, Pearson's *r*), indicating how they were calculated

Our web collection on statistics for biologists contains articles on many of the points above.

Software and code

Policy information about availability of computer code

Data collection	MS spectra from MALDI-TOF MS were collected from Data Explorer software 4.11 and Flex Control software 2.4.<br>MS and MS/MS spectra from LC-MS were collected from Xcalibur 3.1 software.
Data analysis	TOF/TOF Series Explorer software 4.1 and Flex Analysis software 3.3 were used to analysis MALDI-TOF MS data.<br>ProteomeDiscoverer 2.1, MASCOT 2.4 and Maxquant 1.6 were applied for database searching of all .raw files.<br>GO analysis was carried out on http://pantherdb.org/ (Panther 15.0) and http://revigo.irb.hr/.<br>Sequence motif analysis was carried out on Weblogo 2.8.2.<br>Origin 8, 3DMAX and Cytoscape 3.7.2 was used for mapping.

For manuscripts utilizing custom algorithms or software that are central to the research but not yet described in published literature, software must be made available to editors and reviewers. We strongly encourage code deposition in a community repository (e.g. GitHub). See the Nature Research guidelines for submitting code & software for further information.

Data

Policy information about availability of data

All manuscripts must include a data availability statement. This statement should provide the following information, where applicable:
- Accession codes, unique identifiers, or web links for publicly available datasets
- A list of figures that have associated raw data
- A description of any restrictions on data availability

The authors declare that all data supplementary the findings of this study are available within the paper and its supplementary information or from the corresponding author upon reasonable request. Proteomics data have been deposited to the ProteomeXchange Consortium [http://proteomecentral.proteomexchange.org] via the iProX partner repository[1] with the dataset identifier PXD017423 (for LB and different carbon source cultured

April 2020

E.coli), PXD021067 (for HeLa and HEPG2).

Field-specific reporting

Please select the one below that is the best fit for your research. If you are not sure, read the appropriate sections before making your selection.

- [x] Life sciences
- [ ] Behavioural & social sciences
- [ ] Ecological, evolutionary & environmental sciences

For a reference copy of the document with all sections, see nature.com/documents/nr-reporting-summary-flat.pdf

Life sciences study design

All studies must disclose on these points even when the disclosure is negative.

Sample size	According to the literature and our estimation of N-phosphoprotein content, about 2.5e7 mammal cells (corresponding to 6 mg total protein) or 12 mg of Escherichia Coli extract were used.
Data exclusions	No data were excluded from the analysis.
Replication	1. For the LC-MS analysis of LB cultured E.coli, 3 replicates were carried out to ensure the reproducibility of the experimental findings. And all attempts at replication were successful.<br>2. For the correlation analysis, 3 replicates were carried out. And all attempts at replication were successful.<br>3. For the experiments involving method establishment, such as SiO2@DpaZn preparation, N-phosphopeptide degradation, N-phosphopeptide recovery, Zeta potential test, etc., 3 replicates were also carried out. And all attempts at replication were successful.<br>4. For the rest of other experiments including the LC-MS analysis of M9 cultured E.coli, HeLa and HEGP2 cells, NMR and ITC experiments, independent experiments were carried out.
Randomization	Not relevant to this study. There was only one cell lysate sample in each experiment.
Blinding	Not relevant to this study. There was only one cell lysate sample in each experiment.

Reporting for specific materials, systems and methods

We require information from authors about some types of materials, experimental systems and methods used in many studies. Here, indicate whether each material, system or method listed is relevant to your study. If you are not sure if a list item applies to your research, read the appropriate section before selecting a response.

Materials & experimental systems

n/a	Involved in the study	
x		Antibodies
	x	Eukaryotic cell lines
x		Palaeontology and archaeology
x		Animals and other organisms
x		Human research participants
x		Clinical data
x		Dual use research of concern

Methods

n/a	Involved in the study	
x		ChIP-seq
x		Flow cytometry
x		MRI-based neuroimaging

Eukaryotic cell lines

Policy information about cell lines

Cell line source(s)	HeLa and Jurkat cell lines were both purchased from American type culture collection (ATCC).
Authentication	None of the cell lines used were authenticated.
Mycoplasma contamination	Cell lines were not tested for mycoplasma contamination.
Commonly misidentified lines (See ICLAC register)	There was no commonly misidentified cell lines used in this study.
